# Long-term potentiation and spatial memory training stimulate the hippocampal expression of RyR2 calcium release channels

**DOI:** 10.3389/fncel.2023.1132121

**Published:** 2023-03-21

**Authors:** Ismael Valdés-Undurraga, Pedro Lobos, Virginia Sánchez-Robledo, Alejandra Arias-Cavieres, Carol D. SanMartín, Genaro Barrientos, Jamileth More, Pablo Muñoz, Andrea Cristina Paula-Lima, Cecilia Hidalgo, Tatiana Adasme

**Affiliations:** ^1^Biomedical Research Institute (BNI), Faculty of Medicine, Universidad de Chile, Santiago, Chile; ^2^IVIRMA, Santiago, Chile; ^3^Center for Advanced Clinical Investigation (CICA), Clinical Hospital, Universidad de Chile, Santiago, Chile; ^4^Clinical Analysis, University Hospital of Salamanca, Salamanca, Spain; ^5^Section of Emergency Medicine, Department of Medicine, Institute for Integrative Physiology, Neuroscience Institute, The University of Chicago, Chicago, IL, United States; ^6^Physiology and Biophysics Program, ICBM, Faculty of Medicine, Universidad de Chile, Santiago, Chile; ^7^Laboratory of Translational Psychiatry, Department of Neuroscience and Department de Psychiatry North, Universidad de Chile, Santiago, Chile; ^8^Translational Neurology Center and Biomedical Research Center, Faculty of Medicine, Universidad de Valparaíso, Valparaíso, Chile; ^9^Institute for Research in Dental Sciences (ICOD), Faculty of Dentistry, Universidad de Chile, Santiago, Chile; ^10^Department of Neuroscience, Faculty of Medicine, Universidad de Chile, Santiago, Chile; ^11^Center for Exercise, Metabolism and Cancer (CEMC), Faculty of Medicine, Universidad de Chile, Santiago, Chile

**Keywords:** ryanodine, theta burst stimulation, Morris water maze, calcium-induced calcium release, synaptic plasticity, spatial memory consolidation

## Abstract

**Introduction:** Neuronal Ca^2+^ signals generated through the activation of Ca^2+^-induced Ca^2+^ release in response to activity-generated Ca^2+^ influx play a significant role in hippocampal synaptic plasticity, spatial learning, and memory. We and others have previously reported that diverse stimulation protocols, or different memory-inducing procedures, enhance the expression of endoplasmic reticulum-resident Ca^2+^ release channels in rat primary hippocampal neuronal cells or hippocampal tissue.

**Methods and Results:** Here, we report that induction of long-term potentiation (LTP) by Theta burst stimulation protocols of the CA3-CA1 hippocampal synapse increased the mRNA and protein levels of type-2 Ryanodine Receptor (RyR2) Ca^2+^ release channels in rat hippocampal slices. Suppression of RyR channel activity (1 h preincubation with 20 μM ryanodine) abolished both LTP induction and the enhanced expression of these channels; it also promoted an increase in the surface expression of the α-amino-3-hydroxy-5-methyl-4-isoxazolepropionic acid (AMPA) receptor subunits GluR1 and GluR2 and caused a moderate but significant reduction of dendritic spine density. In addition, training rats in the Morris water maze induced memory consolidation, which lasted for several days after the end of the training period, accompanied by an increase in the mRNA levels and the protein content of the RyR2 channel isoform.

**Discussion:** We confirm in this work that LTP induction by TBS protocols requires functional RyR channels. We propose that the increments in the protein content of RyR2 Ca^2+^ release channels, induced by LTP or spatial memory training, play a significant role in hippocampal synaptic plasticity and spatial memory consolidation.

## 1. Introduction

Neuronal Ca^2+^ signals generated *via* Ca^2+^ release from the endoplasmic reticulum (ER) play a significant role in hippocampal synaptic plasticity, learning, and memory processes (Baker et al., 2013; Paula-Lima et al., 2014). Previous reports have shown that the rat hippocampus expresses mainly the type-2 (RyR2) ryanodine receptor (RyR) isoform, with lower expression levels of the type-3 RyR (RyR3) and the IP_3_R type-1 (IP_3_R1) isoforms (Adasme et al., 2011); it does not contain measurable protein levels of the RyR1 isoform whereas RyR1 mRNA levels, albeit detectable, are close to the detection limit of qPCR (Adasme et al., 2011; Arias-Cavieres et al., 2017; More et al., 2018a).

Different stimulation protocols or diverse memory inducing procedures enhance the expression of Ca^2+^ release channels in hippocampal neuronal cells or tissue. High-frequency field stimulation of primary hippocampal cultures, through activation of RyR-mediated Ca^2+^-induced Ca^2+^ release (CICR) increases the protein content of the RyR2 isoform (Riquelme et al., 2011), as does incubation with gabazine (Lobos et al., 2021), an inhibitor of GABA_A_ receptors that promotes spontaneous hippocampal neuronal activity (Pegoraro et al., 2010; Mauceri et al., 2011; Bengtson et al., 2013). Likewise, incubation of primary hippocampal cultures with brain-derived neurotrophic factor (BDNF) or injection of BDNF into the rat hippocampus increase the protein contents of RyR2 and of the RyR3 and the IP_3_R1 isoforms (Adasme et al., 2011). The BDNF-induced increase in RyR2 protein content in primary hippocampal neurons involves the activities of ERK1/2, nitric oxide synthase, and NADPH oxidase (NOX-2) and is prevented by N-Acetylcysteine (More et al., 2018a).

Electrophysiological experiments have revealed that sustained induction of long-term potentiation (LTP) for 1 h increases RyR2, RyR3, and IP_3_R1 protein levels in hippocampal slices from young but not from aged rats; in contrast, induction of long-term depression (LTD) for 1 h does not modify the hippocampal levels of these three proteins in young rats (Arias-Cavieres et al., 2017). In addition, training rats in the Morris water maze (MWM) to promote spatial learning increases RyR2 mRNA and protein levels in rat hippocampus (Zhao et al., 2000; Adasme et al., 2011), as does rat exposure to the Oasis maze protocol (More et al., 2018a) or to fear conditioning protocols (More et al., 2018b). Training rats in the MWM also increases the hippocampal protein contents of the RyR3 and the IP_3_R1 channel isoforms (Adasme et al., 2011), whereas nicotine administration to mice upregulates RyR2 levels in the cortex and ventral midbrain, two brain areas associated with cognition and addiction (Ziviani et al., 2011). Moreover, after performing a long-term object location task—but not a novel object recognition task—young rats display higher RyR2, RyR3, and IP_3_R1 protein contents in the hippocampal CA1 region relative to the controls (Arias-Cavieres et al., 2017). In addition, spatial training and exposure to an enriched environment increase hippocampal RyR3 mRNA levels and that Mecp2—a DNA methylation reader with a critical role in experience-dependent plasticity—acts as a RyR3 transcriptional activator, which contributes to experience-dependent plasticity (Torres et al., 2017).

Here, we report that LTP induced by theta-burst stimulation (TBS) of the CA3-CA1 hippocampal synapse produced significant increments in RyR2 mRNA and protein levels. Preincubation with ryanodine—at concentrations that abolish RyR-mediated Ca^2+^ release without affecting ER calcium content (Adasme et al., 2015)—prevented these enhancements. Incubation of hippocampal slices with inhibitory ryanodine also produced a small but significant reduction in dendritic spine density and promoted an increase in the surface expression of the α-amino-3-hydroxy-5-methyl-4-isoxazolepropionic acid (AMPA) receptor subunits GluR1 and GluR2. We also report here that training rats in the MWM induced the persistent expression of the RyR2 channel isoforms for several days after the end of the training period.

## 2. Materials and methods

### 2.1. Materials and antibodies

The FD Rapid GolgiStain Kit was from FD Neurotechnologies (Columbia, MD). Anti-GluR1 (monoclonal), anti-GluR2 (monoclonal), anti-RyR3 (polyclonal), and PVDF membranes were from Merk Millipore (Darmstadt, Germany). TRIzol reagent, digestion (DNA-free Kit), DNAase (Turbo DNA-free^TM^ kit), were from Ambion, Invitrogen (Carlsbad, CA); Improm II^TM^ reverse transcriptase from Promega (Madison, WI; Brilliant III Ultra-Fast SYBR^®^ Green QPCR Master Mix was from Agilent Technologies (Santa Clara, CA); Mounting solution for slide coating was from Vectashield (Vector Laboratories, Burlingame, CA). Protease inhibitors (Calbiochem, La Jolla, CA, USA), Anti-RyR2 antibodies (monoclonal), NeutrAvidin Plus and sulfo-NHS-LC-biotin were from Pierce-ThermoFisher (Waltham, MA). Monoclonal anti-β actin (A5316), horseradish peroxidase-conjugated anti-rabbit IgG, and horseradish peroxidase-conjugated anti-mouse IgG were from Sigma-Aldrich (St. Louis, MO).

### 2.2. Hippocampal slice preparation

Male Sprague Dawley rats (P28–31) under isofluorane anesthesia were euthanized by decapitation, and their brains were quickly removed. The hippocampus was dissected in cold dissection buffer containing (in mM: 212.7 sucrose, 5 KCl, 1 MgCl_2_, 2 CaCl_2_, 10 glucose, 1.25 NaH_2_PO_4_, 26 NaHCO_3_, pH 7.4) and was cut into 400 μm transversal slices using a vibratome (Vibratome 1000 plus, Ted Pella Inc., Redding, CA). Hippocampal slices were transferred to an immersion storage chamber kept at room temperature in artificial cerebrospinal fluid (ACSF) containing (in mM: 124 NaCl, 5 KCl, 1.25 NaH_2_PO_4_, 1 MgCl_2_, 2 CaCl_2_, 10 glucose, 26 NaHCO_3_, pH 7.4), in 95% O_2_/5% CO_2_. Incubations with ryanodine were subsequently performed for 1 h at 32°C. After pharmacological manipulations, slices were exposed to LTP-inducing protocols, frozen in liquid nitrogen and stored at –80°C for subsequent biochemical analysis. All experimental procedures used in this work complied with the "Guiding Principles for Research Involving Animals and Human Beings" of the American Physiological Society and were approved by the Bioethics Committee on Animal Research, Faculty of Medicine, Universidad de Chile.

### 2.3. Electrophysiological determinations

All experiments were performed in an immersion-recording chamber. Hippocampal slices were superfused with ACSF (in 95% O_2_/5% CO_2_) at a rate of 2 ml/min at 30 ± 2°C. Field excitatory postsynaptic potentials (fEPSP) were evoked by square current pulses (0.2 ms) delivered with a concentric bipolar stimulating electrode (FHC Inc., Bowdoinham, ME) located in the Schaeffer collateral–commissural fibers; fEPSP were recorded using glass microelectrodes (2–3 MΩ) filled with ACSF placed into the *stratum radiatum* of the CA1 region. To evaluate presynaptic components of the responses, two pulses were applied every 15 s, with inter-stimulus intervals starting at 20 ms and ending at 640 ms, doubling the interval after each trial. The results are presented as the ratio between the initial fEPSP slopes evoked by the second stimulus over the first. After monitoring pre-synaptic responses, LTP was evaluated by adjusting the stimulus intensity to generate fEPSPs values corresponding to half of the maximal evoked response, with pulses applied every 15 s until a stable baseline was attained at least for 20 min. To induce LTP, the TBS protocol was applied, consisting of four trains of 10 bursts at 5 Hz each (1 burst = 4 pulses at 100 Hz; Arias-Cavieres et al., 2017). In all experiments, fEPSP recordings were continued for 60 min after applying the TBS protocol. Recordings were filtered at 10 kHz and were digitized at 5 kHz, using Igor Pro (WaveMetrics Inc., Lake Oswego, OR). Synaptic responses, quantified as the initial slope of the evoked fEPSPs, were plotted as percentage of the basal change relative to the slope of the baseline records (adjusted to 100%).

### 2.4. Morris water maze (MWM) training

Behavioral training and testing were conducted in a circular pool (diameter 100 cm, depth 40 cm) maintained at a temperature of 22 ± 2°C. In the first session, which consisted of three trials performed for three consecutive days, male rats (2–3 months) were allowed to swim for 2 min in the pool (Control-Free Swim). During the training session (Invisible Mobile platform), consisting of three sessions per day at 3–4 h intervals performed for six consecutive days, a 10 cm wide circular platform was present 1 cm under the water level; the platform placement in each quadrant was changed in each training session. The learning session was performed on six consecutive days and consisted of three sessions per day, performed at 3–4 h intervals. For the learning sessions, the escape platform was rendered invisible by placing it 1 cm below the water surface without changing its position in the third quadrant. The animals were allowed to swim and find the invisible platform for 1 min. The spatial memory test was performed 3, 10 or 19 days after the end of the training period. To this aim, rats were allowed to swim for 1 min in search of the quadrant where the platform was located during the training session. Rat behavior was recorded with a video camera positioned over the behavioral apparatus, and the collected videos were analyzed with the ANY-MAZE software (Stoelting Co., Wood Dale, IL). A scheme illustrating the training and memory tests is illustrated in Supplementary Figure 1.

### 2.5. RNA extraction from hippocampal slices and qRT-PCR analysis

Two hippocampal slices per condition were homogenized in 100 μl Trizol. After complete homogenization with an electric homogenizer, 900 μl Trizol were added, followed by addition of 200 μl chloroform. Samples were vortex-shaken, incubated on ice for 3 min and centrifuged for 15 min at 12,000× *g* at 4°C. The aqueous phase was collected, and after addition of 500 μl isopropanol and 2 μl GlycoBlue, samples were stored at V80°C overnight. The following day, samples were thawed at 4°C and centrifuged for 25 min at 12,000× *g*; the supernatants were removed and 500 μl of 70% ethanol was added to wash the pellets. The resulting suspensions were centrifuged again for 15 min at 12,000× *g* at 4°C; this pellet washing procedure was repeated five times. The resulting pellets were resuspended in 20 μl of nuclease-free water; to remove any contaminating traces of genomic DNA, a DNAase digestion step was performed. To quantify RNA levels, the 260/280 absorbance ratio was measured. Subsequently cDNA was synthesized from a total of 1 μg of RNA using a reverse transcriptase kit. For amplification, 25 ng of cDNA was used in a final volume of 20 μl. Amplification was performed using the primers shown in Table 1. Quantitative real-time PCR (qRT-PCR) was performed on the amplification system using the SYBR Green DNA-binding probe. The mRNA levels of the RyR2 and RyR3 isoforms were calculated with the relative 2^−ΔΔCt^ method (Pfaffl 2001) and values were normalized by β-actin mRNA levels. The purity of the products was verified by analyzing the dissociation curves. All samples were analyzed at least in triplicate.

**Table 1:** Primer sequences used.

Gene	Forward (5’- 3’)	Reverse (5’- 3’)
RyR2 (LTP)	CTACTCAGGATGAGGTGCGA	CTCTCTTCAGATCAAAGCCA
RyR3 (LTP)	GAAGCCTGTTGGTGGACCATA	TCCAGAGTGTTTGCATAAAGGAG
RyR2 (MWM)	AATCAAAGTGGCGGAATTTCTTG	TCTCCCTCAGCCTTCTCCGGTTC
RyR3 (MWM)	AGAAGAGGCCAAAGCAGAGG	GGAGGCCAACGGTCAGA
β-actin (LTP/MWM)	TCTACAATGAGCTGCGTGTG	TACATGGCTGGGGTGTTGAA

### 2.6. Western blot analysis

The hippocampal tissue was placed in a glass/Teflon homogenizer and 200 μl of lysis solution (in mM: 20 MOPS-Tris, pH 7.0, 300 sucrose, 2 EDTA; 1% NP-40 and 0.1% SDS) were added, plus 1 mM BAPTA and protease inhibitors. After complete homogenization, samples were incubated on ice for 10 min, sonicated three times for 20 s and centrifuged at 600× *g* for 20 min at 4°C. The supernatants were collected, and each sample was separated into several aliquots that were stored frozen at −80°C. After protein determination by a turbidimetric method, samples were denatured at 50°C for 20 min in reducing buffer solution (34.8% glycerol, 1 M Tris-Base, 2 mM EDTA, 0.1 M dithiothreitol (DTT), 8% SDS, 0.04% Bromophenol Blue). Samples were loaded on 3.5–8% polyacrylamide/Tris-acetate gradient gels, containing 15% a polyacrylamide basal layer; electrophoresis was performed in Tris-Tricine buffer system for 4 h at constant 80 volts. Proteins were transferred from the gels to PVDF membranes with Tricine-Bis Tris Propane transfer buffer (in mM: 12.5 Tricine, 12.5 Bis Tris Propane, 1 EDTA) plus 10% methanol for 2.5 h at constant 350 mA. The PVDF membranes were incubated overnight at 4°C with 5% milk blocking buffer and were subsequently incubated at room temperature with specific antibodies as detailed in Figure legends. The membranes were revealed with a chemiluminescence system. The resulting images were scanned and analyzed using the ImageJ program.

### 2.7. Golgi-Cox staining of rat hippocampal slices

Staining was performed with the FD Rapid Golgi Stain kit (Creative Biolabs, Shirley, NY). Slices (200 μm thick) were incubated in impregnating solution (kit A + B solution in 1:1 ratio) for 3–4 days at 37°C in the dark. Next, the impregnating solution was replaced by kit solution C and after incubation at room temperature in the dark for 3 days, the slices were transferred to gelatin-coated slides. After successful adherence, the slices were washed two times for 4 min with Milli-Q water and a few drops of a mixture of kit solutions D, E and Mlilli-Q water were added (1:1:2 ratio, respectively). After incubation for 10 min, the slices were washed two times for 4 min with Milli-Q water and were dehydrated for 4 min in 50% ethanol, 4 min in 75% ethanol, 4 min in 95% ethanol and four times in absolute ethanol. The dehydrated slices were rinsed with Histoclear (three washes, 4 min each), and mounted with Dibutylphthalate Polystyrene Xylene (DPX) medium (Ranjan and Mallick, 2012; Zhao et al., 2013). Images of the slices were acquired with a 100× objective with an additional optical magnification of 1.5. A minimum of three slices per condition were analyzed, and at least 10 neurites were analyzed for each slice. To be considered for analysis, each neurite had to belong to a neuron with a visible soma, reside in the CA1 zone of the hippocampus (postsynaptic) and in the same plane; within this plane, the extension of the neurite had to be between 20–50 μm in length. The z-stacks were acquired with the Nikon Nis Elements software (Nikon Instruments Inc, Melville, NY), for subsequent blind analysis using the ImageJ program.

### 2.8. Determination of GluR1 and GluR2 surface expression

Hippocampal slices, were washed three times with ice-cold ACSF, transferred to an incubation plate with 3 ml of ACSF, and incubated over ice for 10 min. After this time, 3 ml of 2× biotin solution [3.6 mg sulfo-NHS-LC-biotin in 0.6 ml HPLC water (6 mg/ml, 20× biotin stock)] was added to obtain a final concentration of 0.3 mg/ml. After incubation over ice for 45 min, the slices were quickly washed three times with cold ACSF and were transferred to another incubation plate containing the biotinylation stop solution (ACSF with 200 mM glycine). After 5 min incubation and two more washes with the stop solution, the slices were washed three times with cold ACSF and were quickly homogenized with 100 μl of extraction buffer (in mM: 1 EDTA, 150 NaCl, 10 Tris–HCl, pH 6.8, 0.2% SDS, 0.5% sodium deoxycholate, 1% Triton X-100, plus 1 mM complete protease inhibitory cocktail (PMSF) and 1 mM 1, 10-phenanthroline). Slices were disrupted by pipetting, followed by 5 min of full power sonication. Samples were incubated next on ice for 10 min and centrifuged for 15 min at 12,000× *g* at 4°C. The supernatants were collected, and the protein concentration was measured (Hartree, 1972). To capture the biotinylated proteins, 100 μg of sample were mixed with 40 μl of NeutrAvidin beads; following incubation for 3 h at 4°C, samples were centrifuged at 3,500× *g* for 1 min, the pellet was collected and washed four times with 500 μl of extraction buffer. Finally, 50 μl of loading buffer were added, heated at 95°C for 5 min, and then the beads were separated from the supernatant by brief centrifugation. Subsequently, to resolve the GluR1 and GluR2 subunits of the AMPA receptor (~100 KDa), the proteins present in the supernatants from homogenized slices or in the supernatants separated from the beads were analyzed by electrophoresis on 7% Tris-glycine polyacrylamide gels, and transferred to PDVF membranes, which were blocked for 1 h at room temperature in Tris buffered saline, containing 0.2% Tween-20 and 5% skim milk. Incubations with primary antibodies, anti-GluR1 and anti-GluR2 (at 1:1,000 dilutions), were carried out overnight at 4°C, followed by incubation with the secondary antibodies conjugated with horseradish peroxidase for 1 h at room temperature. The membranes were revealed using the ECL kit and automated image captures were made with the ChemiDoc system, which were later analyzed with the imageJ program.

### 2.9. Statistical analyses

Statistics were performed using Prism 5 (GraphPad Software, Inc.). Comparisons between two groups were conducted using unpaired two-tailed *t*-test with Welch’s correction or paired comparisons, where appropriate. Unless otherwise stated, data are presented as mean ± S.E.M; where appropriate, individual responses are overlaid over the mean. Significance was defined as *p* < 0.05. Further details are provided in Figure legends.

## 3. Results

In this work, we induced LTP in hippocampal slices (CA3-CA1) from male rats by applying TBS protocols and measured the mRNA levels and protein contents of RyR2 and RyR3 channels after 1 h of stimulation. We also tested the effects of abolishing RyR activity with inhibitory ryanodine on RyR2 and RyR3 expression before and after applying the TBS protocol, and on dendritic spine density and the surface expression of the GluR1 and GluR2 AMPA receptor subunits. In addition, we determined several days after training rats on the Morris water maze the protein contents of the RyR2 and RyR3 channel isoforms.

### 3.1. LTP induction increased RyR2 mRNA and protein levels

**Figure 1:**
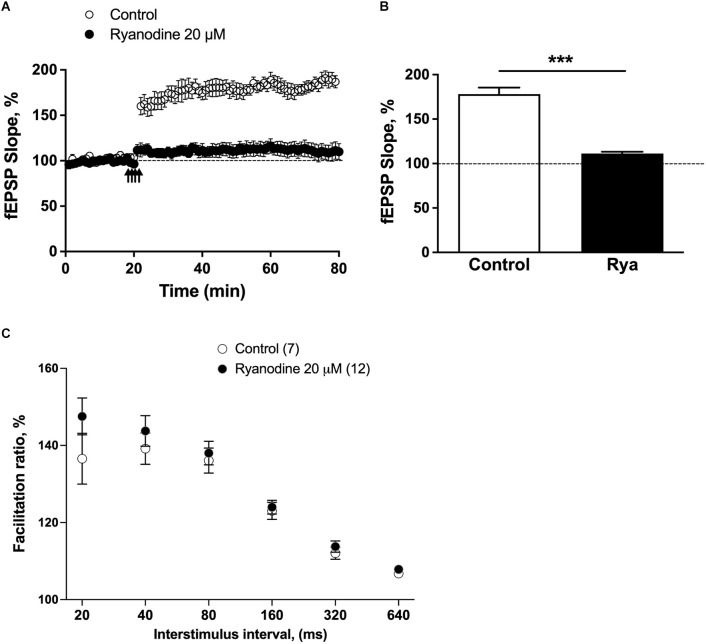
Inhibitory ryanodine suppressed LTP without changing release probability. **(A)** Average fEPSP slope values, normalized to the basal values before TBS (100%), were plotted as a function of time for control slices and for slices pretreated for 1 h with an inhibitory concentration of ryanodine (20 μM). The statistical analysis was performed *via* unpaired t-test with Welch’s correction; ***p < 0.001. **(B)** The fEPSP slopes values displayed 60 min after TBS are presented as the percentage of change from baseline in control slices (*N* = 7 slices; four animals) and slices preincubated for 1 h with 20 μM ryanodine (Rya; *N* = 7 slices; four animals). The statistical analysis was performed with Mann-Whitney test; ***p < 0.001. **(C)** The paired-pulse ratio was defined as the slope of the fEPSP evoked by the second stimulus divided by the slope of the initial fEPSP slope evoked by the first stimulus at each interval. Each data point represents the mean paired-pulse ratio per group; values represent mean ± SEM. The analysis was performed by GraphPad Prism software using two-tailed ANOVA; differences were not statistically significant.

To induce LTP, we used the TBS protocol (four pulses at 100 Hz repeated with 200 ms inter-burst-intervals). As illustrated in Figure 1A, four trains of TBS effectively induced the LTP response, which remained constant for the entire 1 h of the recording period. Pretreatment of slices with 20 μM ryanodine for 1 h before applying the TBS protocol suppressed the LTP response (Figures 1A,B) but it did not affect the paired-pulse response (Figure 1C), as previously reported (Arias-Cavieres et al., 2017). Therefore, these results support previous reports indicating that RyR inhibition does not affect the generation of presynaptic local Ca^2+^ signals involved in the fast paired-pulse response (Raymond and Redman, 2006; Galeotti et al., 2008; Baker et al., 2013; Brini et al., 2014; Paula-Lima et al., 2014; Arias-Cavieres et al., 2018; More et al., 2018b), but prevents LTP induction by disrupting Ca^2+^-dependent postsynaptic pathways.

**Figure 2:**
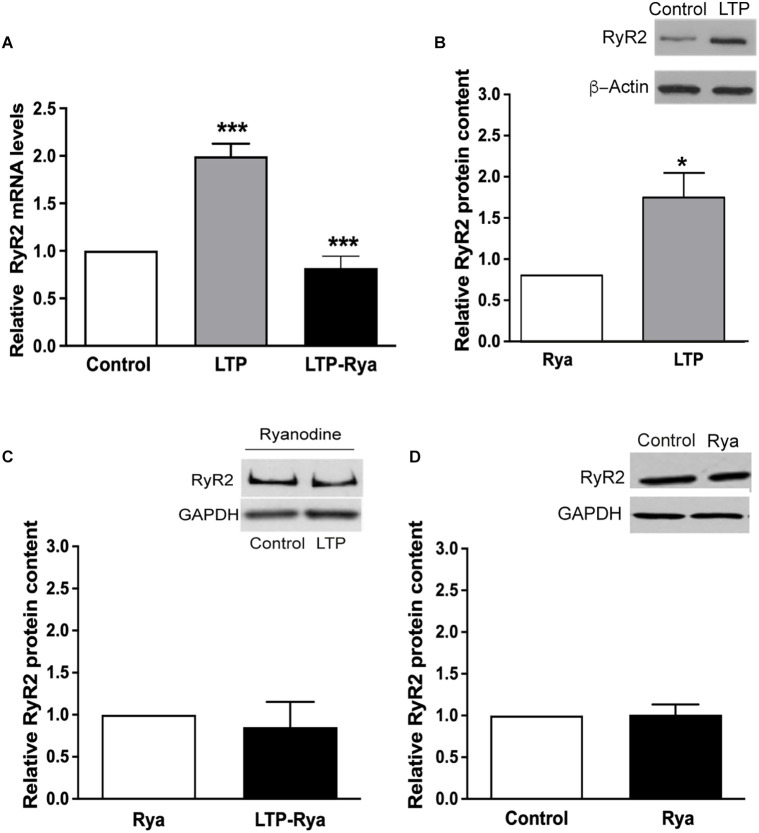
Inhibitory ryanodine suppressed the LTP-induced increases in RyR2 mRNA levels and protein content. **(A)** qRT-PCR analysis of RyR2 mRNA levels determined 60 min after applying the TBS protocol to control slices or to slices pretreated for 1 h with 20 μM ryanodine. The results are expressed as Mean ± SEM (*N* = 4); ***p < 0.001. **(B)** RyR2 protein contents were determined 60 min after applying the TBS protocol to control slices or to slices preincubated for 1 h with 20 μM ryanodine. The results are expressed as Mean ± SEM (*N* = 4); *p < 0.05. **(C)** RyR2 protein contents were determined in slices pretreated for 1 h with 20 μM ryanodine; values were determined after 60 min in slices exposed only to basal stimulation or to the TBS protocol. **(D)** RyR2 protein contents were determined in unstimulated slices; results illustrate the RyR2 protein contents in control slices or in slices preincubated for 1 h with 20 μM ryanodine. In **(C)** and **(D)**, the results are expressed as Mean ± SEM, (*N* = 4); differences were not statistically significant. The statistical analysis was performed with one-way ANOVA followed by Tuckey’s multiple comparisons *post-hoc* test **(A)** and with two-tailed Student’s paired *t*-test **(B,C,D)**.

The induction of LTP for 1 h, produced significant increments in RyR2 mRNA (Figure 2A) and protein levels (Figure 2B), determined in slices that displayed significant LTP induction for 60 min (Supplementary Figure 2). In contrast, slices preincubated for 1 h with 20 μM ryanodine did not present the RyR2 mRNA and protein increases displayed by control slices after LTP induction (Figures 2A,B). Slices preincubated with for 1 h with 20 μM ryanodine displayed the same RyR2 protein contents regardless of the application or the TBS protocol (Figure 2C). Likewise, incubation of unstimulated slices for 1 h with inhibitory ryanodine did not modify RyR2 protein content relative to control slices (Figure 2D), indicating that RyR channel blockade did not modify basal RyR2 protein levels.

Sustained LTP induction for 1 h by four trains of TBS did not modify the levels of RyR3 mRNA, determined in the same slices, but caused a moderate increase in RyR3 protein content, which did not reach statistical significance (Supplementary Figure 3). Preincubation with 20 μM ryanodine prior to applying the TBS protocol did not modify RyR3 mRNA levels relative to the control values and suppressed the modest RyR3 protein increase displayed by control slices after LTP induction (Supplementary Figure 3).

### 3.2. Training rats in the Morris water maze caused a sustained increase in RyR2 protein content

**Figure 3:**
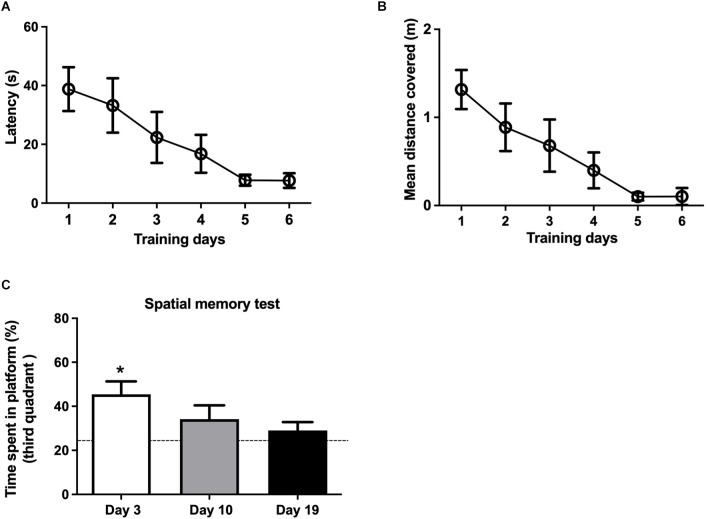
Effects of spatial memory training on memory consolidation as a function of time. **(A)** The escape latencies and **(B)** the mean distances covered were measured during the three sessions performed in each training day for three different groups of rats; data represent the average values displayed by these three groups. **(C)** Time spent in the third quadrant, measured 3, 10 and 19 days after the end of the training period. Three days after training, rats stayed for significantly longer times in the third quadrant respect to free swim control rats (dotted line). Values represent Mean ± SEM (*N* = 3); *p < 0.05, determined by one-way ANOVA followed by Tuckey’s multiple comparisons *post-hoc* test.

Three separate groups of rats, trained in the MWM for 6 days (Supplementary Figure 1), displayed significant reductions in latency (the time to find the platform), which decreased from 45 s at day 1 to less than 10 s at day 6 (Figure 3A). Training also decreased the mean distance covered in all three groups (Figure 3B).

To assess memory consolidation, the three independent groups of rats were tested 3, 10, or 19 days after the end of the training period. The group of rats tested 3 days after training displayed a significant preference for the third quadrant (Figure 3C), which contained the platform during training, indicating that the training protocol used here induced spatial memory consolidation. The independent group tested 10 days after training also displayed a preference for the third quadrant; however, these values did not reach statistical significance. Rats tested 19 days after training did not display significant memory consolidation (Figure 3C).

**Figure 4:**
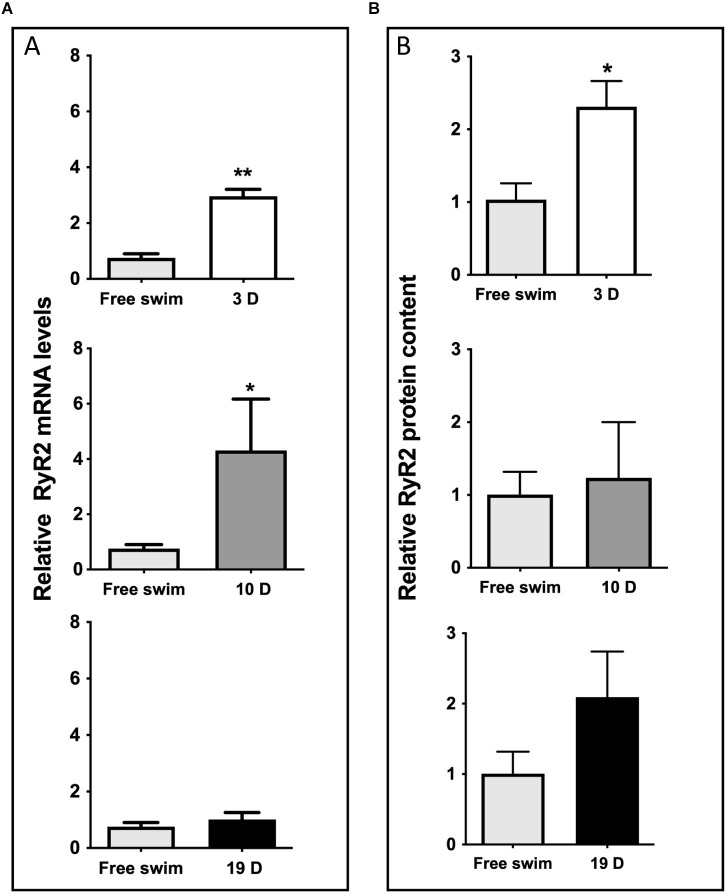
Spatial memory training increased RyR2 mRNA levels and protein content. **(A)** qRT-PCR analysis of RyR2 mRNA expression levels, normalized with β-actin and obtained from the whole hippocampal tissue after the free swim sessions or 3, 10 or 19 days after the end of the training period. The bars represent the analysis of a group of *N* = 6 free swim rats; *N* = 5 rats were evaluated after 3 days; *N* = 5 rats were evaluated after 10 days and *N* = 4 rats were evaluated after 19 days. Values represent Mean ± SEM; **p < 0.005. Results were analyzed with unpaired Mann-Whitney test. **(B)** RyR2 protein contents in hippocampal tissue collected after the free swim sessions 3, 10 or 19 days after the end of the training period; β-actin was used as loading control. Densitometry analysis of Western blots revealed significant increments in RyR2 protein content 3 days after training. Values represent Mean ± SEM (*N* = 4 for RyR2 evaluated 3 and 19 days after training; *N* = 3 for RyR2 evaluated 10 days after training). *p < 0.05, determined by two-tailed Mann Whitney test.

Spatial memory consolidation generated persistent increments in hippocampal RyR2 mRNA levels (Figure 4A), and RyR2 protein content (Figure 4B), which remained significantly elevated 3 days after the completion of the training period (2.10 ± 0.41, *N* = 5), compared to the average values displayed by hippocampal samples from the free swim controls (1.01 ± 0.31, *N* = 6). The RyR2 protein contents remained somewhat increased 10 days after training (1.24 ± 0.77, *N* = 4), and 19 days after training (2.10 ± 0.65, *N* = 3) when compared to the control values, albeit these values did not reach statistical significance. A representative image of the CA1 region immunostained for RyR2 (Supplementary Figure 3A) illustrates the substantial increase in RyR2 protein content displayed by the CA1 region 3 days after the end of the training period relative to the free swim controls. A representative Western blot image (Supplementary Figure 3B) corresponds to the results presented in Figure 4B. In contrast, RyR3 protein content did not present significant increments when measured during the consolidation phase (Supplementary Figure 4).

### 3.3. Effects of incubation of rat hippocampal slices with inhibitory ryanodine on dendritic spine density and GluR1 and GluR2 expression

**Figure 5:**
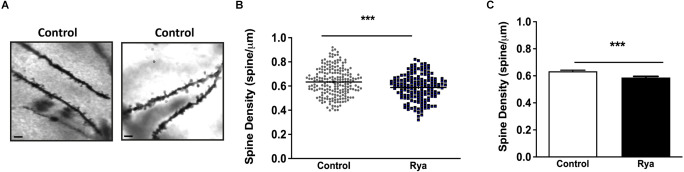
Inhibitory Ryanodine decreased dendritic spine density in the hippocampal CA1 region. **(A)** Representative images of neurites from the CA1 region of rat hippocampal slices stained with FD rapid Golgi Stain. Scale Bar: 2 μm. **(B)** Spine density analysis was performed by calculating the ratio between the number of spines and the dendritic length (in μm). Spine density values displayed by neurites in control slices (circles; *N* = 79 slices, 211 neurites) or neurites in slices incubated for 1 h with 20 μM ryanodine (squares; *N* = 71 slices, 160 neurites). A total of 7,775 spines were counted in this study. Horizontal bars represent the mean values in each case. **(C)** Spine density values displayed by control slices or by slices preincubated for 1 h with 20 μM ryanodine. Statistical analysis was performed by Student’s paired *t*-test, ***p < 0.001 (*N* = 5 animals).

To visualize dendritic spines, hippocampal slices (200 μm) were fixed with paraformaldehyde and subjected to Golgi-Cox staining. As illustrated in Figure 5, slices incubated with inhibitory ryanodine (20 μM) for 1 h displayed a minor (≈10%) but significant reduction in dendritic spine density.

**Figure 6:**
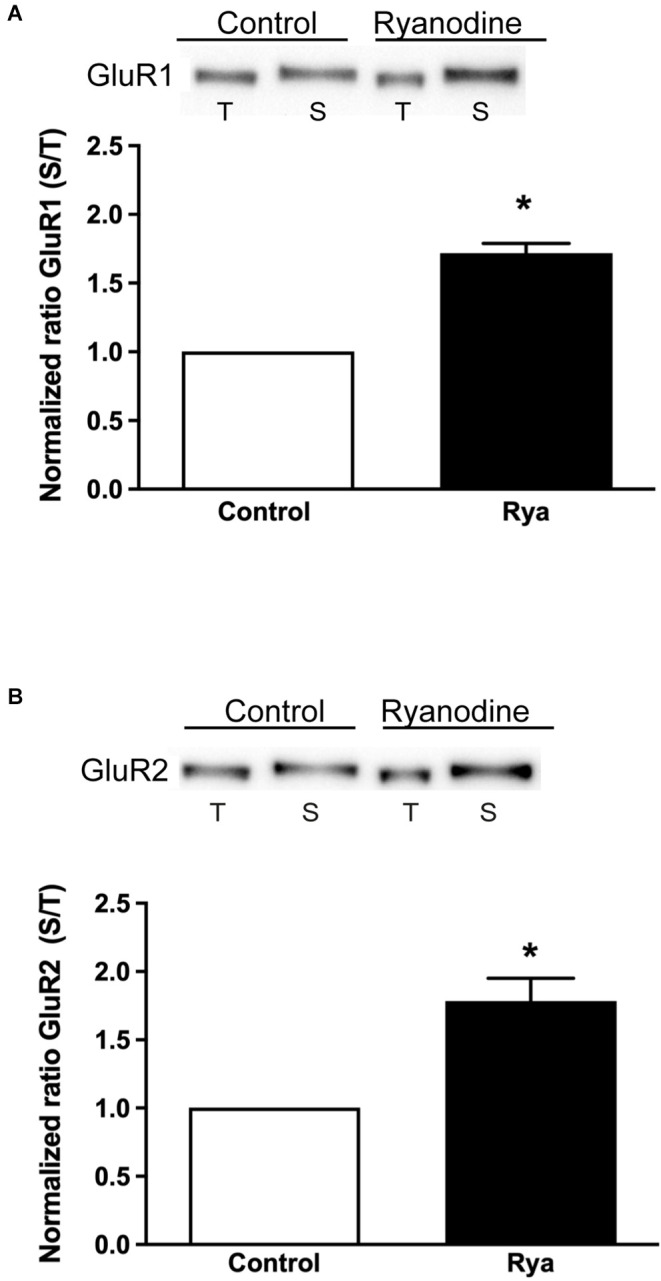
Inhibitory ryanodine increased the surface expression of GluR1 and GluR2. The GluR1 and GluR2 surface membrane protein contents were normalized by the total amount of these subunits present in 20 μg of protein extract. **(A,B)** Comparison between the normalized contents of GluR1 and GluR2 at the surface (S) vs. their total content (T). Protein contents were determined in control slices and in slices incubated for 1 h with 20 μM ryanodine; the ratio between the surface and the total content displayed by control slices was given a value equal to 1. Values are expressed as Mean ± SEM. Statistical analysis was performed by Student’s t-test for a single sample. *p < 0.01.

In addition, we evaluated if inhibitory ryanodine modified the expression of surface AMPA receptors. To this aim, we assessed the membrane surface levels of the GluR1 and GluR2 subunits, which integrate most hippocampal AMPA receptors (Lu et al., 2009; Schwenk et al., 2014), using protein biotinylation assays (Kim and Kovacs, 2011). We found that incubation of slices for 1 h with inhibitory ryanodine (20 μM) induced a significant increase in the surface expression of both the GluR1 and the GluR2 AMPA receptor subunits (Figure 6).

## 4. Discussion

Neuronal Ca^2+^ signals generated by Ca^2+^ entry *via* plasma membrane Ca^2+^ channels and Ca^2+^ release from the endoplasmic reticulum (ER) are required for hippocampal synaptic plasticity and spatial memory processes (Verkhratsky and Shmigol, 1996; Raymond and Redman, 2006; Galeotti et al., 2008; Baker et al., 2013; Brini et al., 2014; Paula-Lima et al., 2014; More et al., 2018b; Hiess et al., 2022). As presented in the Introduction section, several studies have revealed that different neuronal stimulation or memory training protocols enhance the hippocampal expression of the ER-resident RyR2 and RyR3 channel isoforms.

### 4.1. The induction of LTP required RyR-mediated Ca^2+^ release and promoted RyR2 expression

Hebbian plasticity, considered at present as the cellular basis of experience-dependent learning and memory processes, comprises bidirectional changes in synaptic strength *via* LTP and LTD (Diering and Huganir, 2018). The dependence of LTP on the increase in intracellular Ca^2+^ levels has been recognized for decades (Malenka et al., 1992; Voronin et al., 1995; Malenka and Bear, 2004). Moreover, as detailed below, RyR-mediated Ca^2+^ release has been implicated in synaptic plasticity through the *in vitro* use of pharmacological agents, which either stimulate or inhibit RyR channel function.

Initial evidence of the possible participation of RyR channels in synaptic plasticity was obtained using hippocampal slices and ryanodine at low concentrations, which activate RyR channels; these conditions induced LTP at low stimulation frequency, which otherwise would have induced LTD (Wang et al., 1996). Furthermore, Ruthenium Red, a non-specific RyR channel inhibitor that also acts as an effective inhibitor of the mitochondrial calcium uniporter (Bernardi and von Stockum, 2012), blocks the LTP response induced by low-frequency stimulation of slices incubated with ryanodine at low concentrations (Wang et al., 1996). Incubation with the RyR inhibitor dantrolene also inhibits LTP induction in the CA1 region of rat hippocampal slices (Obenaus et al., 1989). However, whereas RyR channels contribute to spontaneous neuronal firing they do not regulate firing activity, since incubation with dantrolene reduces the peak amplitude of spontaneous transients without altering firing frequency (Gavello et al., 2018). It is likely that only strong stimulation protocols, but not the stimulation protocols used to trigger basal synaptic transmission, engage RyR-mediated calcium release. In addition, RyR channel inhibition by sustained preincubation with μM ryanodine before tetanic stimulation also suppresses long-lasting hippocampal LTP but not the induction of LTP by HFS protocols, whereas RyR channel activation by low ryanodine concentrations converts early LTP into late LTP (Lu and Hawkins, 2002). Likewise, incubation with low ryanodine concentrations to stimulate RyR channel activity converts short-term hippocampal potentiation into LTP (Grigoryan et al., 2012). Most of these previous studies have used HFS-type stimulation protocols to induce LTP (Lu and Hawkins, 2002; Sajikumar et al., 2009), except for a report describing that incubation of slices for 10 min with 10 μM ryanodine decreased LTP induction after applying one TBS train, but not four or eight TBS trains (Raymond and Redman, 2002). However, it is worth noting that ryanodine at ≥10 μM concentrations binds preferentially to open RyR channels, so long incubation periods with inhibitory ryanodine are required to effectively abolish RyR activity.

The present work adds to the above list of studies that have analyzed the participation of Ca^2+^ release from intracellular reservoirs, and, more specifically the contribution of RyR-mediated Ca^2+^ release, to the LTP response (Wang et al., 1996; Shimuta et al., 2001; Lu and Hawkins, 2002; Raymond and Redman, 2002; Sajikumar et al., 2009). Here, we report that applying a rigorous RyR channel blocking protocol—1 h preincubation of slices with 20 μM ryanodine—suppressed the hippocampal CA1 LTP response induced by four TBS trains. Hence, we conclude that the Ca^2+^ entry signals are insufficient to trigger the signaling cascades required for the induction of LTP by four TBS trains. Accordingly, we conclude that amplification of the initial Ca^2+^ entry signals by RyR-mediated CICR is required to stimulate downstream Ca^2+^-dependent signaling pathways that mediate LTP induction. Further studies are required to identify the nature of these pathways, which are likely to engage Ca^2+^-dependent postsynaptic proteins that mediate the increased hippocampal fEPSP response induced by TBS.

In this work we also present novel evidence indicating that after 1 h of sustained LTP responses induced by four TBS trains, rat hippocampal slices display a significant increment in RyR2 mRNA and protein levels. Future studies should assess whether these rather fast increments in RyR2 mRNA and protein content represent *de novo* transcription and translation, mRNA and protein stabilization, or both mechanisms. We also detected a modest increase in RyR3 protein content, which did not reach statistical significance, plus undetectable changes in RyR3 mRNA levels. Except for the lack of increase in RyR3 levels, these results—obtained in whole hippocampal slices—are in concordance with a previous study, where we reported increased levels of RyR2 and RyR3 mRNA and protein levels in the CA1 region after 1 h of LTP-induction by TBS protocols (Arias-Cavieres et al., 2017). Possibly, RyR3 mRNA and protein levels increase preferentially in the CA1 region after LTP induction, so that determinations performed in whole hippocampal slices do not evidence these increments. In addition, we describe in this work that RyR activity suppression with 20 μM ryanodine prevented the RyR2 increase induced by LTP. We have reported that neuronal stimulation promotes RyR-dependent Ca^2+^ signals that reach the nucleus, where they promote an increase in RyR2 mRNA levels (Lobos et al., 2021). Here, we present additional evidence indicating that the significant neuronal stimulation induced by the TBS protocol increased RyR2 mRNA levels and RyR2 protein content, and that these increments required functional RyR channels.

### 4.2. Suppression of RyR-mediated Ca^2+^ release produced a modest decrease in dendritic spine density in hippocampal slices, and increased GluR1 and GluR2 surface location

Synaptic activity and intracellular Ca^2+^ signals strictly modulate dendritic spine formation, plasticity, and maintenance (Fifková, 1985; Oertner and Matus, 2005; Vlachos et al., 2009; Adasme et al., 2011; More et al., 2018b). Here, we show that inhibitory ryanodine caused a modest but significant reduction in dendritic spine density in hippocampal slices. Hence, we propose that basal RyR channel activity is directly or indirectly necessary for the maintenance of the basal dendritic spine density displayed by unstimulated hippocampal slices.

Our results also show that the surface membrane levels of the GluR1 and GluR2 subunits of the AMPA receptor significantly increased in slices pre-incubated with inhibitory ryanodine. These increments may reflect neuronal attempts to compensate for the decrease in Ca^2+^ signaling pathways provoked by the inhibition of RyR-mediated Ca^2+^ signals, since surface AMPA receptors play a central role in hippocampal synaptic transmission and LTP induction (Shimuta et al., 2001; Goswami et al., 2017; Diering and Huganir, 2018; Pereda et al., 2019). Future studies should investigate the specific neuronal pathways engaged in these responses.

### 4.3. Spatial memory consolidation increased the expression of RyR2 Ca^2+^ release channels

Previous reports have shown that neuronal cell stimulation promotes Ca^2+^ entry mediated by voltage or agonist-activated plasma membrane Ca^2+^ channels; through CICR the resulting cytoplasmic Ca^2+^ signals stimulate Ca^2+^ release from the ER, amplifying the initial signal and triggering neuronal responses necessary for processes involved in memory and learning (Balschun, 1999; Shimuta et al., 2001; Galione and Churchill, 2002; Mellentin et al., 2007; Galeotti et al., 2008; Adasme et al., 2011; Baker et al., 2011; Lü et al., 2012; Hopp et al., 2014). Here, we report that spatial memory consolidation produced a significant increase in RyR2 mRNA and protein contents when tested 3 days after the end of training; these levels were increased even after 10 or 19 days after training, albeit at these later times the RyR2 increments did not reach significance.

Several studies have implicated Ca^2+^ release through RyR channels in learning and memory processes (Edwards and Rickard, 2006; Galeotti et al., 2008; Baker et al., 2010; Adasme et al., 2011; More et al., 2018b; Hiess et al., 2022). Previous studies reporting increments in RyR2, RyR3, and IP_3_R1 protein content induced in rat hippocampus by spatial memory training, object location or fear conditioning protocols (Zhao et al., 2000; Adasme et al., 2011; Arias-Cavieres et al., 2017; More et al., 2018a, b) suggest that these enhancements have a role in memory processes. Our earlier findings, showing that stimulation of RyR-mediated Ca^2+^ release induced by intrahippocampal injection of ryanodine at agonist concentrations boosts spatial memory formation and consolidation (Adasme et al., 2011), support the proposal that increasing RyR protein content, and thus RyR activity, is important for hippocampal memory formation and consolidation. However, an experimental test of this proposal presents serious limitations, since it would be necessary to eliminate specifically the increments in RyR2 protein content induced by spatial memory training without modifying RyR2 basal levels, which are required for memory formation.

## 5. Conclusions

The present results show that hippocampal LTP induction by four TBS trains requires functional RyR channels and that both TBS-induced LTP and spatial memory training in the MWM enhance the hippocampal expression of RyR2 Ca^2+^ release channels. These results further implicate RyR2-mediated Ca^2+^ release as a central participant in hippocampal neuronal function.

## Data availability statement

The original contributions presented in the study are included in the article/Supplementary materials, further inquiries can be directed to the corresponding author.

## Ethics statement

The animal study was reviewed and approved by Ethical Commitee for research in animals, Faculty of Medicine, Universidad de Chile, Santiago, Chile.

## Author contributions

TA, CH, and AP-L supervised and funded this work, designed experiments, analyzed the experimental results, and wrote the manuscript. IV-U performed and analyzed all LTP experiments. PL performed and analyzed spatial training and qPCR experiments, analyzed results, and contributed to manuscript writing. VS-R performed spatial training and Western blot experiments. AA-C supervised, designed and carried out spatial memory experiments, and contributed to manuscript writing. CS performed and analyzed qPCR experiments, analyzed results. GB supervised and performed LTP experiments. JM performed Western blot experiments and analyzed results. PM supervised and designed LTP experiments, analyzed the experimental results. All authors contributed to the article and approved the submitted version.
