# Supplementary material for: Long-term potentiation and spatial memory training stimulate the hippocampal expression of RyR2 calcium release channels
Source: Front Cell Neurosci. 2023 Mar 21;17:1132121. doi: 10.3389/fncel.2023.1132121 (PMC10071512; doi:10.3389/fncel.2023.1132121)
Supplement: Supplementary file 1 [file Data_Sheet_1.PDF]

Supplementary Figure 1

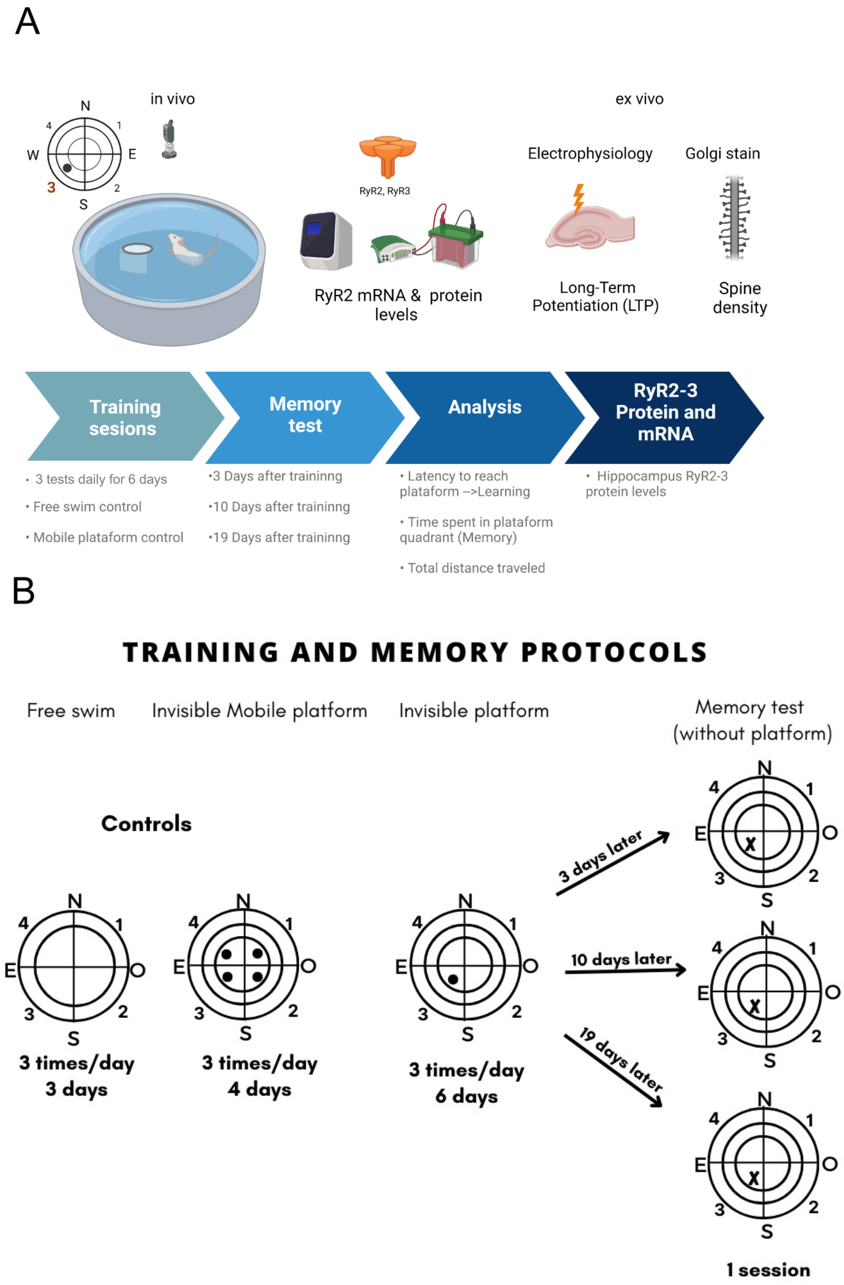

Supplementary Figure 1. A) Scheme illustrating the protocols used in this study. **B)** Training and Memory testing protocols, including all internal controls for the spatial memory test.

Supplementary Figure 2

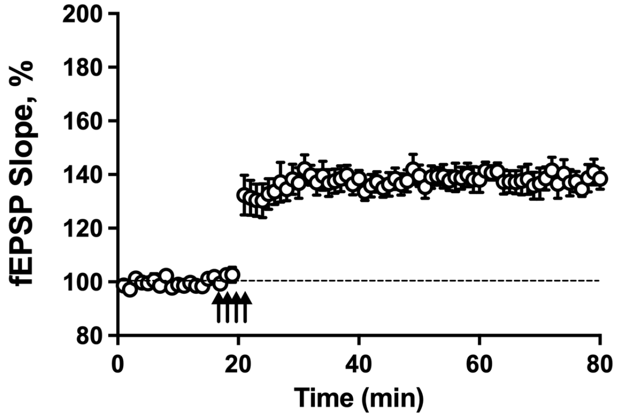

Supplementary Figure 2. LTP response. The graph illustrates the average LTP responses induced by TBS (4 trains), of the slices (N = 4) analyzed for the RyR2 determinations illustrated in Figure 2.

Supplementary Figure 3

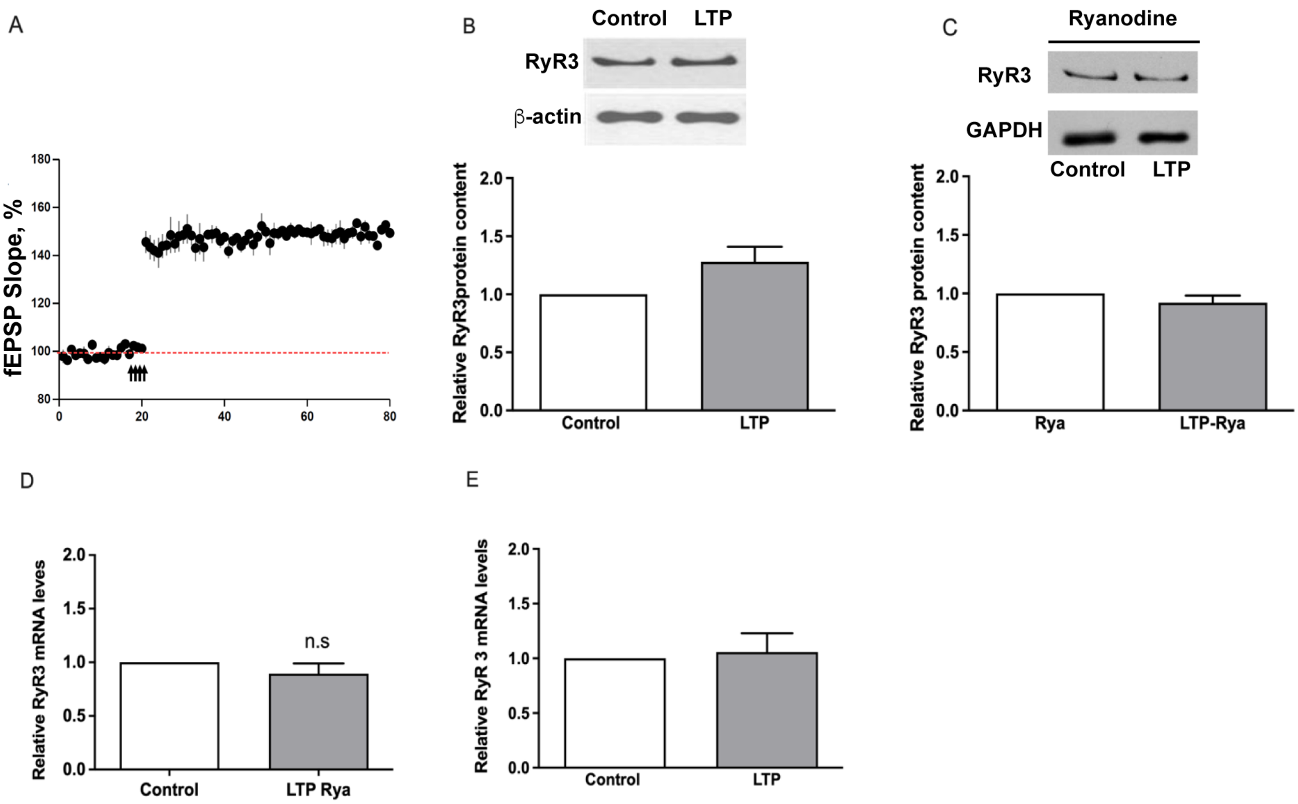

Supplementary Figure 3. Determination of RyR3 mRNA levels and protein contents in control slices and after TBS. A) Representative graph of the mean fEPSP slope values plotted as a function of time and normalized to the basal slope values (100%) displayed by slices before applying the TBS protocol. B) & C) RyR3 protein contents determined 60 min after applying the TBS protocol to control slices or to slices pretreated for 1 h with 20 µM ryanodine. D) & E) qRT-PCR analysis of RyR3 mRNA levels determined 60 min after applying the TBS protocol to control slices or to slices pretreated for 1 h with 20 µM ryanodine. The results are expressed as Mean ± SEM (N = 3). The differences between values did not reach statistical significance when tested with the two-tailed Student's paired t-test.

Supplementary Figure 4

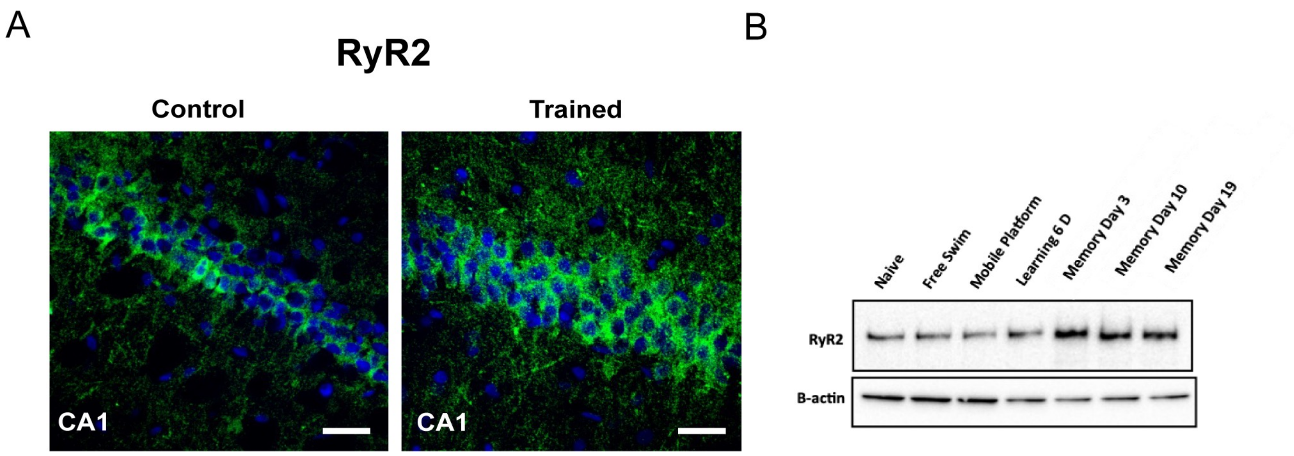

Supplementary Figure 4. A) Representative images of RyR2 immunofluorescence, detected in the CA1 region of hippocampal slices obtained the third day after the end of the training period in the MWM. RyR2 immunodetection (green) was determined with specific RyR2 antibodies; nuclei were stained with Hoechst (blue). Scale bar 20 µm . B) Representative Western blot showing RyR2 protein content in the whole hippocampus, determined at different stages of memory training or after consolidation.

Supplementary Figure 5

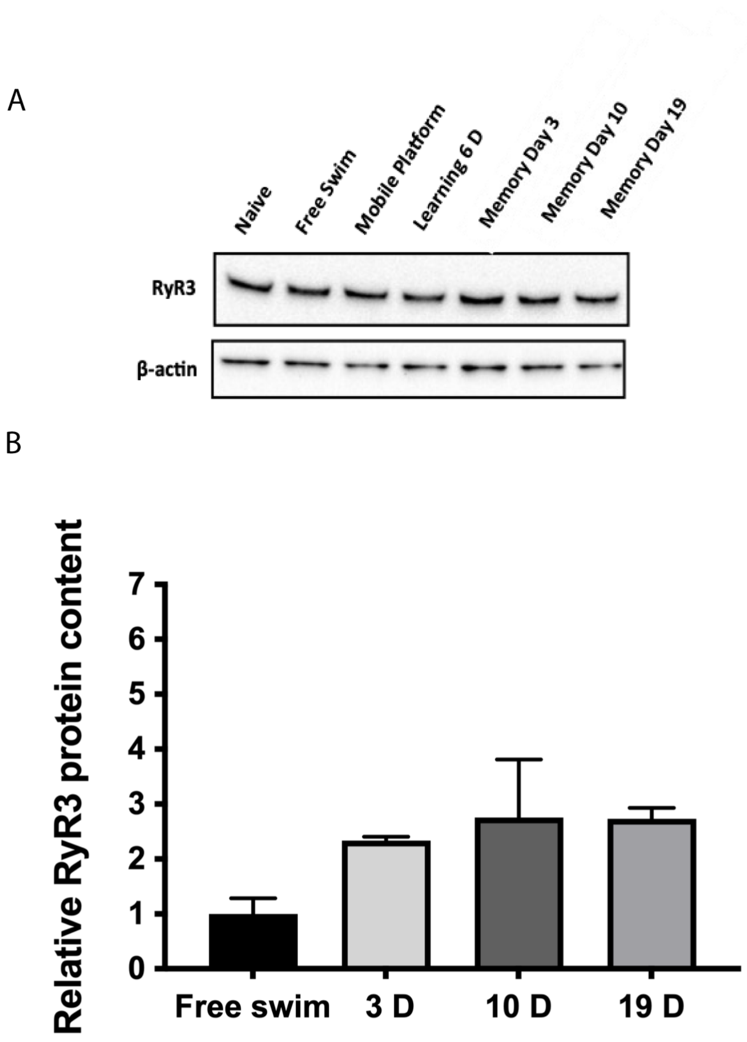

Supplementary Figure 5. RyR3 protein contents displayed by hippocampal slices after training rats in the MWM. A) Representative Western blot showing RyR3 protein contents determined at different stages of memory training or after consolidation. B) RyR3 protein contents in hippocampal tissue collected after the free swim sessions or 3, 10 or 19 days after the end of the training period; β-actin was used as loading control. Densitometry analysis of Western blots revealed that the values (Mean ± SEM, N = 3) of RyR3 protein contents, determined 3, 10 and 19 days after training did not reach statistical significance after analysis with the two-tailed Mann Whitney test.
